# Effect of Disease-Associated Germline Mutations on Structure Function Relationship of DNA Methyltransferases

**DOI:** 10.3390/genes10050369

**Published:** 2019-05-14

**Authors:** Allison B. Norvil, Debapriya Saha, Mohd Saleem Dar, Humaira Gowher

**Affiliations:** Department of Biochemistry, Purdue University, West Lafayette, IN 47907, USA; anorvil@purdue.edu (A.B.N.); saha27@purdue.edu (D.S.); darm@purdue.edu (M.S.D.)

**Keywords:** ADCA-DN, HSAN1E, TBRS, dwarfism, DNMT3A, DNMT1, rare diseases, PCC/PGL, DNA methylation

## Abstract

Despite a large body of evidence supporting the role of aberrant DNA methylation in etiology of several human diseases, the fundamental mechanisms that regulate the activity of mammalian DNA methyltransferases (DNMTs) are not fully understood. Recent advances in whole genome association studies have helped identify mutations and genetic alterations of DNMTs in various diseases that have a potential to affect the biological function and activity of these enzymes. Several of these mutations are germline-transmitted and associated with a number of hereditary disorders, which are potentially caused by aberrant DNA methylation patterns in the regulatory compartments of the genome. These hereditary disorders usually cause neurological dysfunction, growth defects, and inherited cancers. Biochemical and biological characterization of DNMT variants can reveal the molecular mechanism of these enzymes and give insights on their specific functions. In this review, we introduce roles and regulation of DNA methylation and DNMTs. We discuss DNMT mutations that are associated with rare diseases, the characterized effects of these mutations on enzyme activity and provide insights on their potential effects based on the known crystal structure of these proteins.

## 1. Introduction

DNA methylation is a highly conserved epigenetic modification in mammals and takes place at the 5′ position of cytosine, largely at the CpG dinucleotide [1,2]. The distribution of DNA methylation in mammalian genomes is bimodal such that the repetitive elements and transposons are most densely methylated and the regions with highest propensity of CpG (CpG islands) are least methylated [3]. DNA methylation increases the information content of the genome through its potential to control gene expression. In regulatory elements of genes, including promoters and enhancers, DNA methylation is largely associated with repressed genes and is often tissue specific. Conversely, high DNA methylation is found in gene bodies of highly transcribing genes. All these observations indicate that interpretation of DNA methylation is dependent on the genomic context. Despite the complexity of DNA demethylation, the active loss of DNA methylation has been observed both during early development and at certain inducible genes in later adulthood [4]. Although a functional demethylase that can directly remove ‒CH3 groups from the 5′C of cytosine has not been discovered, reversal of DNA methylation can be mediated by the conversion of methyl to hydoxymethyl and higher oxidation states by the Tet family of methylcytosine dioxygenases. This can lead to progressive loss of the modification because these oxidized states unlike DNA methylation cannot be maintained. Besides being an intermediate of DNA demethylation process, hydroxymethylation at regulatory elements, which are in the *primed state*, alter the signal output of DNA methylation by changing its detectability [5,6,7,8].

DNA methyltransferases (DNMTs) are a class of enzymes that catalyze the transfer of a methyl group from S-Adenosyl-L-methionine (AdoMet) to DNA. Mammalian DNMTs belong to two structurally and functionally distinct families, DNMT1 and DNMT3 [9,10,11]. In somatic cells, 60–80% of CpG sites are methylated and DNMT1 diligently copies the methylation patterns from the parent to the daughter strand post-replication and repair [1,12,13]. This activity particularly ensures the maintenance of unmethylated regions by averting spurious de novo DNA methylation. The DNMT3 family includes DNMT3A and DNMT3B, which are the de novo methyltransferases, and one regulatory factor, DNMT3-Like protein (DNMT3L) [11]. DNMT3L is catalytically inactive but interacts with both DNMT3A and 3B to enhance their enzymatic activity [14]. The DNMT3 proteins are required for the establishment of genomic DNA methylation during embryogenesis after its erasure at the preimplantation stage [13]. Whereas DNMT1 is ubiquitously expressed, DNMT3 enzymes show distinct tissue specific expression and methylate the regulatory elements of the transcriptionally inactive genes. Several lines of evidence including distinct phenotype of DNMT3A and DNMT3B KO mice indicate distinct biological functions for these enzymes [15]. Studies of molecular and cellular phenotypes resulting from DNMT mutations have largely contributed to our understanding of the biological roles of DNA methylation [16].

Mammalian DNMTs constitute a C-terminal catalytic domain, a structure which is highly conserved from bacteria to humans [9,17]. Given mammalian DNMTs have weak sequence specificity, their target site recognition is guided by the N-terminal regulatory region, which interacts with transcription factors, chromatin binding proteins, and histone-tail modifications [18]. Consequently, these interactions regulate site-specific DNA methylation leading to differential gene expression. Abnormal patterns of DNA methylation have been observed in several diseases and in all types of cancer. This could be caused by either loss of function of DNMTs or their interactions with modulators [19,20]. Recent high throughput sequencing have revealed mutations in DNMTs associated with several diseased states. Interestingly, in all reported disorders only one of the three enzymes accumulates mutations, leading to distinct phenotypes [21]. Although these mutations are distributed throughout the *DNMT* gene, most of them tend to cluster in the functional domains of these enzymes. The adverse effect of these mutations on the catalytic activity and function of DNMTs has been established in multiple reports [22,23,24,25,26]. The following content will discuss recent advancements in the investigation of etiological consequences of germline–transmitted mutations in DNMT1 and DNMT3A and the effect of these mutations on catalytic and targeting mechanism of the enzymes.

## 2. Structural and Functional Alterations of DNMT1 by Disease Associated Mutations

**Figure 1:**
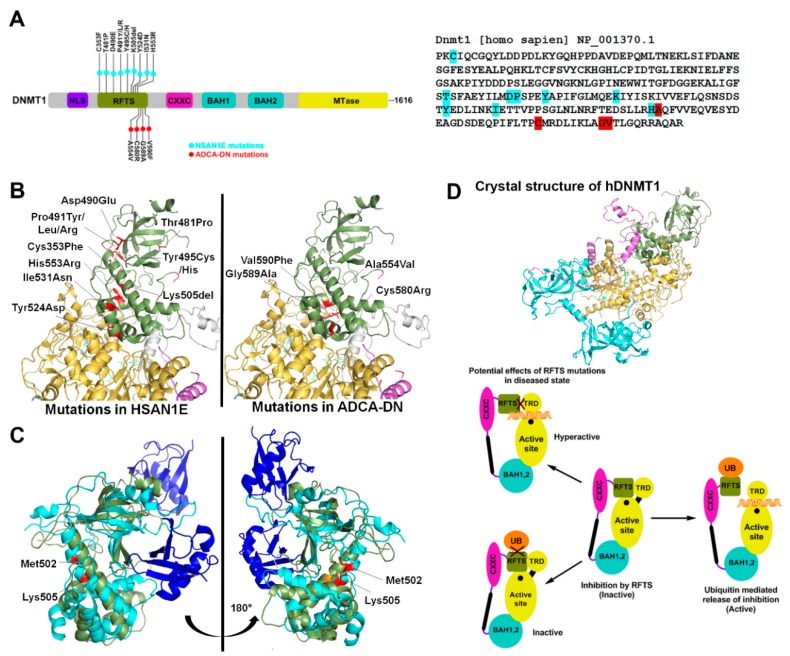
HSAN1E (hereditary sensory neuropathy with dementia and hearing loss) and ADCA-DN (autosomal dominant cerebellar ataxia, deafness and narcolepsy) mutations in DNMT1 (**A**) Left Schematic representation of the *hDNMT1* gene. HSAN1E mutations are listed above the gene in light blue, while ADCA-DN mutations are listed below the gene in red. Right Nucleotide sequence of the RFTS (replication foci-targeting sequence) domain, with the mutations highlighted in color corresponding to the schematic. (**B**) Crystal structure of hDNMT1 (351–1600) from the Protein Data Bank (PDB: 4WXX). The cartoon structure of the RFTS domain is green, the CXXC domain is purple, and the MTase (methyltransferase) domain is yellow. All disease mutations are located in the RFTS domain, and are shown as stick structures in red. The positions of HSAN1E and ADCA-DN mutations are shown in the left and right DNMT1 structure respectively. (**C**) Overlay of the hDNMT1 RFTS domain bound (light blue) and unbound (green) to two molecules of ubiquitin (dark blue) from the PDB: 4WXX and 5YDR. When ubiquitin is bound, the RFTS domain bends about 30° at Met502. The HSAN1E mutation Lys505del and Met502 are shown in red in RFTS domain bound to ubiquitin and in orange in the unbound RFTS domain. (**D**) Model showing the effect of mutations in the RFTS domain on the catalytic mechanism of DNMT1. DNMT1 is auto-inhibited by the interaction of its RFTS domain with the target recognition domain (TRD) in MTase domain that prevents DNA binding. When RFTS interacts with ubiquitin, auto-inhibition is released allowing TRD to interact with the hemi-methylated DNA. However, mutations in the RFTS that alter its binding to ubiquitin will prevent enzyme activation, while mutations that alter its binding to the TRD will leave the enzyme in a hyperactive state.

The eukaryotic DNMT1 is a multimodular protein comprising of a replication foci-targeting sequence (RFTS), a DNA binding CXXC domain, two bromo-adjacent homology (BAH) domains and a C-terminal catalytic domain (Figure 1A) [27]. DNMT1 has an intrinsic preference for hemimethylated CpG sites. This preference is further modulated by interactions of both CXXC and RFTS domains with the DNA binding region of DNMT1 leading to autoinhibition. The CXXC domain binds to unmethylated CpG dinucleotides and sandwiches a section of highly acidic amino acids (the autoinhibitory BAH1-CXXC linker) between the DNA and DNMT1 active site. The autoinhibition by RFTS is relieved by its interaction with UHRF1 (ubiquitin-like, containing PHD and RING finger domains 1), which binds to hemimethylated CpG dinucleotides. It is suggested that hemimethylated DNA from UHRF1 is transferred to the active site of DNMT1 after the inhibitory RFTS has been displaced by the UHRF1/hemimethylated DNA complex [28]. The multiple functional domains of the N- terminus have different roles including coordination of methylation and replication during S-phase, partial suppression of de novo methylation and nuclear localization [2,11,29].

Exome sequencing studies revealed several mutations in DNMT1 that result in two adult onset, progressive neurological disorders. These mutations are germline dominant and include 13 amino acid substitutions in the RFTS domain that potentially disrupt the catalytic activity of the enzyme (Figure 1A). The first is hereditary sensory neuropathy with dementia and hearing loss (HSAN1E) and the second is autosomal dominant cerebellar ataxia, deafness and narcolepsy (ADCA-DN), caused by progressive loss of sensory neuron function [30,31,32].

### 2.1. HSAN1E

To date, nine heterozygous mutations of the *DNMT1* gene have been identified in HSAN1E patients. These mutations are located mostly in exon 20, which encodes part of the RFTS domain (Figure 1A). The first discovered mutations include substitution of two contiguous amino acids Asp490Glu and Pro491Arg, and a Tyr495Cys substitution. Systematic investigation revealed that these mutations lead to protein degradation, reduced DNMT1 activity and defective binding to heterochromatin in G2 phase [32]. This ultimately leads to widespread DNA hypomethylation including pericentromeric satellite 2 sequences, other repetitive elements, intergenic regions, imprinted genes, and transcriptional start sites. At some CpG islands, site-specific hypermethylation was also reported [33]. The mechanism explaining hypermethylation can be interpreted from a study in cancer cells showing that the deletion of RFTS domain makes DNMT1 hyperactive and available for euchromatic binding. This was suggested to be due to loss of RFTS interaction with an unknown heterochromatin binding protein leading to aberrant localization. A point mutation that could have a similar effect on RFTS’s interaction with heterochromatin could potentially cause hypermethylation at CpG islands. [34]. Studies in mouse embryonic stem cells (ESCs) revealed that DNMT1 mutations Pro491Tyr and Tyr495Cys lead to decreased binding with the E3 ubiquitin ligase UHRF1. The ESCs overexpressing the variant DNMT1 enzymes failed to properly differentiate into neuronal progenitor cells, suggesting a differentiation defect as a possible mechanism for disease progression [35]. In addition to the aforementioned mutations, six other mutations in RFTS domain were identified in HSAN1E patients (Figure 1A) [30,36,37,38]. However, in absence of their biochemical characterization, the role of several of these mutations in disease development is not understood. Based on crystal structure analysis, we speculate that these mutations may lead to altered domain structure or interfere with protein-protein interactions (Figure 1B) [39]. The UHRF1 interaction region of DNMT1 spans from residues 458–500, suggesting that Thr481Pro mutation may also cause decreased UHRF1 binding. Biochemical studies however show no effect of this mutation on its localization to replication foci [30]. UHRF1 interacts with DNMT1 through its N-terminal UBL (ubiquitin like) domain. Crystal structure of RFTS domain co-crystalized with ubiquitin shows interaction with two ubiquitin molecules [40]. To accommodate the binding of two ubiquitin molecules, the RFTS domain undergoes a drastic conformational change, bending an α helix by about 30° at Met502 (Figure 1C) [40]. Deletion of Lys505 may prevent the conformational change and affect the RFTS-ubiquitin interaction [40] (Figure 1C). His553, which is located in exon 21, interacts with Glu504 and Lys505 and may facilitate this conformational change, which can be affected by His553Arg mutation [38] (Figure 1C). Based on its position in RFTS domain, the Cys353Phe substitution may perturb zinc binding in the RFTS domain and/or affect protein stability (Figure 1B). Indeed, a study using recombinant expression of many of these variants showed cytosolic aggregation and early degradation of the GFP-tagged mutant proteins [30]. Given that, DNMT1 protein is present in appreciable levels in neurons, cellular toxicity caused by protein aggregates, may underlie some clinical manifestations [30]. Together these studies support the conclusion that loss of DNMT1 targeting causes site-specific changes in DNA methylation in the HSAN1E patients.

### 2.2. ADCA-DN

ADCA-DN patients are reported to have four missense substitutions, Ala554Val, Cys580Arg, Gly589Ala, and Val590Phe. Similar to HSAN1E, all four mutations map to the RFTS domain of DNMT1, however they occur exclusively in exon 21 [31,37,41]. All four mutations are located in the α helical bundle of the RFTS C-lobe, which has a hydrophobic pocket at the center. Therefore substitutions, Ala554Val and Val590Phe, present in the hydrophobic pocket and Gly589Ala, closely located to the hydrophobic pocket, may destabilize the RFTS domain (Figure 1B). Further, these substitutions could also impair autoinhibition by weakening the interaction of the RFTS C-lobe with the DNA binding region of the MTase domain, rendering the enzyme hyperactive with the potential to be mistargeted (Figure 1D). This speculation is supported by data showing that the truncation of RFTS domain leads to dysregulation of DNMT1 activity [34]. Methylation profiling of ADCA-DN patients showed global hypomethylation and hypermethylation specifically at around 80 CpG islands of which nearly half were associated with promoters and rest were inter- or intragenic. The differentially methylated regions were enriched in genes for cellular and anatomical developmental processes [42]. However, the effect of these changes on expression of associated genes and consequent biological function is unknown.

Besides HSAN1E and ADCA-DN disorders, previous work has established the role of aberrant DNA methylation in neurological disorders, such as Alzheimer’s and Parkinson’s disease [43,44,45,46,47]. However, the effect of aberrant DNA methylation is potentially due to mis-regulation of DNMTs and/or interactions of methyl-CpG-binding domain (MBD) proteins, such as, MeCP2 with methylated DNA [48]. Mutation of DNMT1 also cause alterations in the genome-wide DNA methylation patterns in colorectal cancer patients [49], however none of the patients with neurological disorders were shown to develop cancer [5,50]. In summary, these studies support that germline versus somatic mutations have a spatiotemporal effect on the activity of DNMT1 during development and adulthood.

## 3. Structural and Functional Alterations of DNMT3 by Disease Associated Mutations

The DNMT3 family consists of two catalytically active DNMTs, DNMT3A and DNMT3B and a catalytically inactive protein, DNMT3L. DNMT3A and DNMT3B have similar domain organization; both have a variable region at the N-terminus, followed by the Pro-Trp-Trp-Pro (PWWP) domain, a Cys-rich Zn-binding domain also called ATRX-DNMT3-DNMT3L (ADD) domain and a C-terminal methyltransferase (MTase) domain [51]. The PWWP domain targets DNMT3A activity by binding to DNA and histone H3 methylated at the Lys36 residue (H3K36me2/3) [52,53,54]. Co-crystal structure of the DNMT3L–ADD domain with histone H3 peptide shows that it specifically interacts with the Lys4 residue only when it is unmethylated (H3K4me0). Methylation of histone H3K4 (H3K4me1/2/3) disrupts this interaction [55]. Interaction of the ADD domain with the DNA binding region of the DNMT3A catalytic domain was revealed in a recent crystal structure suggesting its role in autoinhibition of the DNMT3A enzymatic activity. This autoinhibition is relieved by the interaction of the DNMT3A-ADD domain with histone H3K4me0 [56]. The dynamic role of this regulatory mechanism was shown to regulate DNA methylation at the enhancers of pluripotency genes during embryonic stem cell differentiation [57].

The MTase domain comprises ten sequence motifs, which are conserved in all cytosine DNMTs and a have direct role in catalysis [9,17]. Motifs I–III are involved in binding to the AdoMet, whereas motifs IV and VI are required for the catalysis. The region between and including motifs VIII and IX is called the target recognition domain (TRD) and is responsible for DNA binding in DNMT3A [58,59]. DNMT3A forms a hetero-tetrameric structure with DNMT3L in which two DNMT3A monomers form the center of the complex, flanked by two DNMT3L monomers on either side [60]. In the heterotetramer of the mouse protein, the DNMT3A–3L interaction is mediated by two Phe residues (261 in DNMT3L and 728 in DNMT3A) and DNMT3A–3A interaction surface comprises Arg881 and Asp872, therefore named as the RD interface [14,60]. In the absence of DNMT3L, DNMT3A forms homo-tetramers and can oligomerize on DNA [26,59]. This property facilitates DNMT3A’s cooperativity, where multiple enzyme units interact with DNA to methylate it at a faster rate [61]. Mutations in the RD interface disrupts DNMT3A DNA binding and activity demonstrating the critical role of protein dimerization in catalysis [26].

While there are only a handful of diseases caused by DNMT3 germline mutations, these diseases are caused by a plethora of mutations. Among all DNMTs, the disease-causing mutations were first discovered in DNMT3B in patients with immunodeficiency, centromeric instability, and facial anomalies (ICF) syndrome [15,62,63]. The implications of these mutations on DNMT3B activity and on the etiology of ICF has been extensively investigated and reviewed [64]. More recently, a high prevalence of DNMT3A somatic mutations were observed in hematological malignancies, Acute Myeloid Leukemia (AML) and Myelodysplastic syndrome (MDS). A series of germline mutations in DNMT3A were discovered in patients with growth syndromes, Tatton-Brown-Rahman syndrome (TBRS) and microcephalic dwarfism (MD). Some of these mutations were also found in hereditary tumors, pheochromocytomas PCC) and paragangliomas (PGL).

### 3.1. Tatton-Brown-Rahman Syndrome

**Figure 2:**
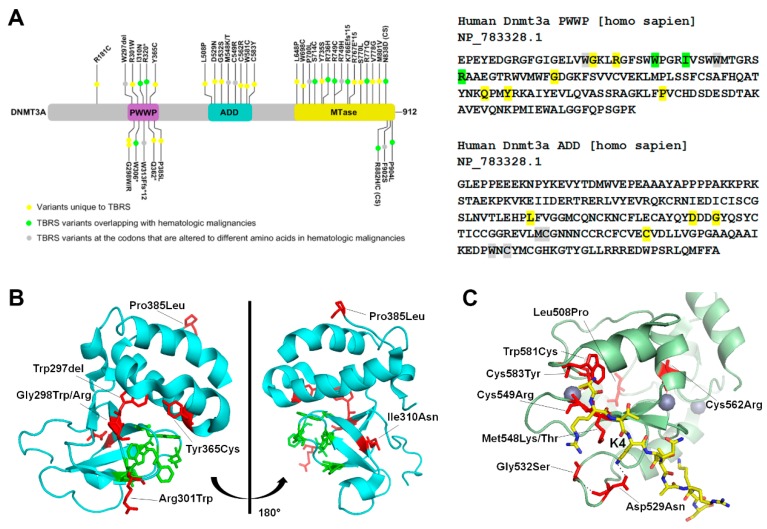
TBRS (Tatton-Brown-Rahman syndrome) mutations in the PWWP and ADD domain of DNMT3A (**A**) Left Schematic representation of the hDNMT3A gene, with mutations listed in different domains. Variants unique to TBRS are highlighted in yellow, TBRS variants overlapping with hematologic malignancies are highlighted in green, and TBRS variants at the codon that are altered to different amino acids in hematologic malignancies are highlighted in grey. In the schematic, (*) is used to indicate a stop codon replacement, Ffs indicates a frame-shift mutation, and CS indicates the catalytic site. Right Nucleotide sequence of the PWWP (Pro-Trp-Trp-Pro) and ADD (ATRX-DNMT3-DNMT3L) domain, with the mutations highlighted in the color corresponding to the schematic. (**B**) Two orientations of the DNMT3A PWWP domain (PDB: 3LLR). The positions of TBRS mutations are shown as stick structures in red, whereas residues part of the aromatic cage that bind to H3K36me2/3 shown as stick structures in green. (**C**) Crystal Structure of the DNMT3A ADD domain (PDB: 4U7T), in green bound to an unmodified H3 peptide shown as a stick structure in yellow. Grey spheres represent bound zinc. The positions of TBRS mutations are shown as a stick structure in red.

DNMT3A related overgrowth syndrome, also known as Tatton-Brown-Rahman syndrome (TBRS) is an autosomal dominant condition characterized by overgrowth, distinctive facial appearance, and intellectual disability. It is caused by heterozygous mutations in DNMT3A that are transmitted through the germ line. In 55 TBRS patients, more than 40 distinct DNMT3A variants have been reported. Of these, most are missense mutations (30 variants), and the rest are nonsense variants, frameshift variants or whole gene deletions [65,66,67,68,69,70,71,72]. The TBRS mutations are specifically localized in each of the three functional domains of DNMT3A, 11 of which overlap with the somatic DNMT3A variants found in hematological malignancies (Figure 2A). A recent study performed a genome-wide DNA methylation analysis of 16 TBRS patients and detailed analysis of the methylation distribution in one patient with Arg771Gln substitution. Their data showed widespread DNA hypomethylation at specific genomic sites located near genes involved in morphogenesis, development, differentiation, and malignancy predisposition pathways, thus providing an important insight into developmental mechanisms that are dysregulated in the disease [73].

In the PWWP domain, several frameshift mutations, one deletion, and five missense substitutions were reported (Figure 2A). Analysis of the crystal structure shows that the missense mutations cluster around the aromatic cage which interacts with H3K36me2/3 [54]. Notable mutations include Arg301Trp, Gly298Trp/Arg, Tyr365Cys and Trp297del, that are near to or interact with the aromatic cage residues, and therefore may disrupt its binding to H3K36me2/3 (Figure 2B).

In TBRS patients, mutations in the ADD domain cluster around the H3K4 binding site. Crystal structure of DNMT3A bound to H3K4me0 shows that Asp529 in the ADD domain makes direct contact with the Lys4 of the histone protein H3 [56]. In absence of the histone H3 peptide, Asp529 also interacts with the DNA binding region, suggesting its involvement in regulation of the autoinhibited state. The TBRS variant Asp529Asn therefore could potentially be hyperactive and mistargeted. Biochemical data showing that the variant Asp529Ala is neither autoinhibited by the ADD domain nor activated by unmethylated H3 peptide support this speculation [56]. Met548 and Trp581 also make direct contact with the H3 tail, and therefore the variants Met548Thr and Trp581Cys may have reduced interaction with histone H3. Indeed, as reported, Met548Trp variant cannot be released from the autoinhibited state in the presence of unmethylated H3 peptide [56]. Besides these mutations Cys549, Cys562, and Cys583 are present in the zinc finger regions. These residues mediate zinc binding and the mutations could potentially alter the DNMT3A structure or stability.

**Figure 3:**
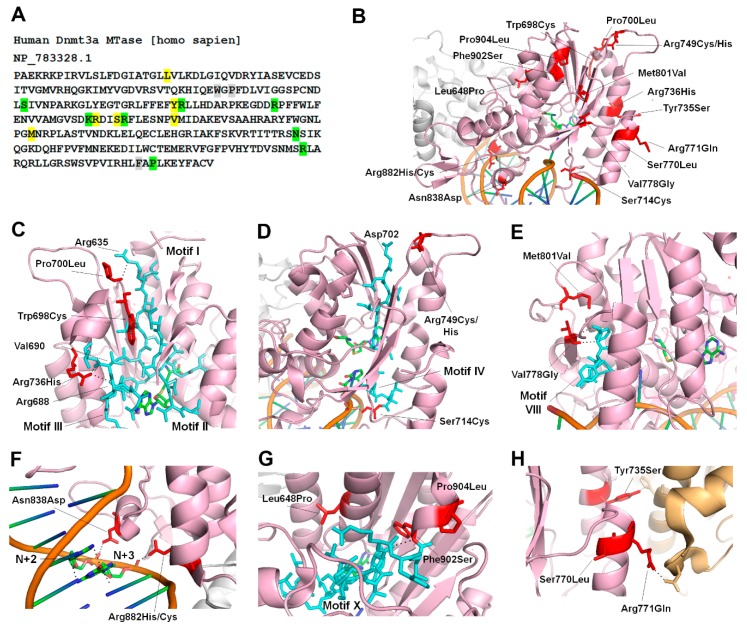
TBRS mutations in the catalytic domain of DNMT3A (**A**) Nucleotide sequence of the hDNMT3A MTase domain. As seen in Figure 2A, variants unique to TBRS are highlighted in yellow, TBRS variants overlapping with hematologic malignancies are highlighted in green, and TBRS variants at the codon that are altered to different amino acids in hematologic malignancies are highlighted in grey. (**B**) Crystal structure of DNMT3A bound to DNA, zoomed in to show only one of the two monomers in the tetrameric structure (PDB: 5YX2). The MTase domain is shown in a pink cartoon structure and the positions of TBRS mutations are shown as stick structures in red. The second monomer of the MTase is shown in grey. (**C**–**G**) Magnified view of the motifs I-III (**C**), motif IV (**D**–**E**), motif VIII (**F**), the TRD (**G**), and motif X (**H**) shown as stick structures in blue and the positions of TBRS mutations shown as stick structures in red. The black dotted lines represent interactions with nearby residues or with the DNA. (**H**) The interface between DNMT3A, pink cartoon, and DNMT3L, orange cartoon. The positions of TBRS mutations shown as stick structures in red. The black dotted lines represent interactions with nearby residues of DNMT3L shown as stick structures in orange.

In TBRS patients, 15 substitutions and 2 frameshift mutations in the MTase domain are distributed in all sequence motifs (Figure 3A) [65,66]. We analyzed the crystal structure of DNMT3A to predict the potential effects of TBRS mutations on its activity (Figure 3B). As visualized in the structure of DNMT3A, Trp698, Pro700 and Arg736, are spatially located near Motifs I–III, and therefore the mutations at these residues could affect the AdoMet binding and catalysis (Figure 3C). Pro700 interacts with Arg635 in Motif I, and Arg736 contacts the backbones of Arg688 in Motif III (Figure 3C). The most recent co-crystal structure of DNMT3A bound to DNA shows that Ser714 interacts with phosphodiester backbone and Arg749 interacts with Asp702 of motif IV, which is involved in catalysis (Figure 3D) [74]. Biochemical investigation of the TBRS substitutions Arg736His and Ser714Cys were recently reported [22]. The data show that Arg736His variant, interestingly, has a 3-fold increase in catalytic turnover but is weakly stimulated by DNMT3L, and has an increased preference for non-CpG sites. However, Ser714Cys has reduced activity as well as reduced stimulation by DNMT3L [22,74]. These data suggest that besides having a direct effect on catalysis, these mutations could alter the structure disrupting the interaction of DNMT3A with DNMT3L. The regions where DNMT3A interacts with the DNA (TRD) spans from motif VIII–IX. Residues that are mutated in or near this region include Val778, Met801, Asn838, Arg882, Phe902, and Pro904. Val778 and Met801 are spatially located near motif VIII, so their mutations to Gly and Val, respectively, may alter DNMT3A’s ability to bind to DNA (Figure 3E). Asn838 interacts with the phosphodiester bond between nucleotides at N+2 and N+3 from the target CpG site, suggesting that the Asn838Asp variant may have a weak binding to DNA (Figure 3F) [59,74]. Phe902, Pro904 and Leu648 are spatially located near motif X (Figure 3G). In vitro studies show that Pro904Leu variant has higher catalytic turnover and negligible effect on DNMT3L mediated DNMT3A stimulation [22].

The TBRS variant, Arg882His, which is also the most prominent somatic variant of DNMT3A in acute myeloid leukemia (AML) patients, has been extensively studied (Figure 3F) [20]. Interestingly mutation of Arg882 is found in 25% AML patients and 25% of TBRS patients indicating this to be a hotspot [72]. Given the majority of TBRS patients are pediatric or young adults, it is difficult to determine the risk of AML, because of its late onset. More recently, two TBRS patients were diagnosed with AML in childhood supporting potential risk and susceptibility of TBRS patients to develop AML [66].

The effect of Arg882His substitution on DNMT3A activity, reported by several studies, caused a 40–80% loss of catalytic activity [75,76]. Arg882 interacts with the phosphodiester bond of the nucleotide at N+3 from the target CpG site. Given the position of R882 close to the RD interface, the mutation disrupts intermolecular interactions, thus preventing the enzyme to form tetramers (Figure 3F). This in turn negatively affects DNMT3A cooperativity and decreases its DNA binding capacity [23]. More recently, the variant was shown to have altered flanking sequence preference around the CpG site [25]. However, it is not clear whether altered flanking sequence preference is a direct consequence of the amino acid substitution or an indirect effect of the loss of cooperativity. Based on its effect on genomic DNA methylation, Arg882His is also suggested to have a dominant-negative effect on the wild type enzyme, however this activity was not confirmed by in vitro experiments [25,76,77].

Another group of TBRS mutations includes residues Tyr735, Ser770, and Arg771, involved in intermolecular interactions with DNMT3L (Figure 3H). Tyr735 and Arg771 make direct contact with His and Asp residues in DNMT3L, respectively, so their mutation may alter the stability of this interface. Biochemical analysis of the Arg771Gln variant interestingly shows an increase in the catalytic activity of the enzyme by 6-fold and no change in the level of stimulation by DNMT3L [22]. However other substitutions of this residue resulted in a decrease in the stimulation by DNMT3L showing the role of this residue in stabilization of DNMT3A-3L interactions.

It is interesting to note that while majority of the mutated residues are highly conserved in DNMT3B, three residues Leu648, Ser714, and Arg736 are conserved in bacterial DNA cytosine methyltransferases including M. HhaI and M. HaeIII, suggesting the effect of substitutions on their conserved structure and catalytic mechanism. However, compared to the level of overlap between DNMT3A mutations found in hematologic malignancies and growth syndromes, very few coincide with mutations of DNMT3B found in ICF patients. Further, the effect of TBRS mutation, Arg736His, on the activity of DNMT3A is notable given that at this position His is normally present in DNMT3B. This suggests that Arg736 in DNMT3A is important for the catalytic mechanism, which has common and distinct features from that of DNMT3B. These observations suggest that the catalytic mechanisms of DNMT3A and DNMT3B are critical for their unique biological functions. While this speculation is anticipated, effects of these substitutions on the catalytic mechanism of DNMT3A compared to DNMT3B need to be further elucidated.

### 3.2. Hereditary Tumors and Microcephalic Dwarfism (MD)

**Figure 4:**
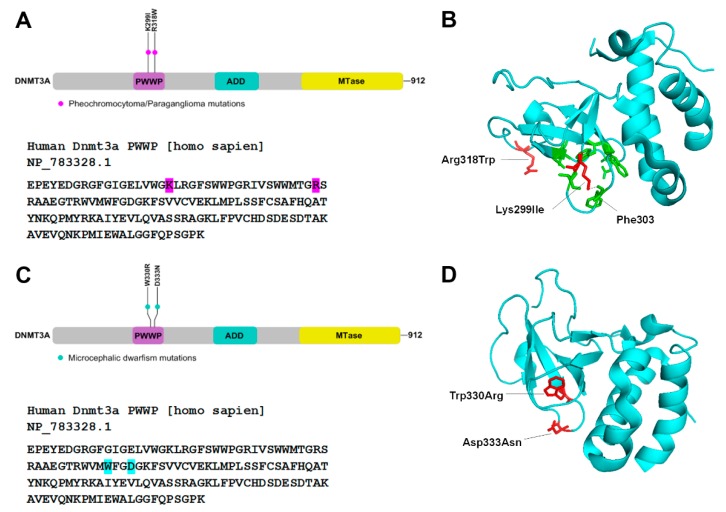
PCC/PGL (Pheochromocytoma/paraganglioma) and MD (microcephalic dwarfism) mutations in PWWP domain of DNMT3A (**A**,**C**) Top Schematic representation of the *hDNMT3A* gene, showing PCC/PGL and MD mutations in the PWWP domain. Below Nucleotide sequence of the PWWP domain, with the mutations highlighted in pink and blue respectively. (**B**,**D**) Crystal structure of DNMT3A PWWP domain shown in blue (PDB: 3LLR). The positions of PCC/PGL mutation (**B**) and MD mutations (**D**) shown as stick structures in red. The aromatic cage residues are shown in green.

Pheochromocytoma/paraganglioma (PCC/PGL) is a rare neuroendocrine malignancy that may develop at various body sites, including the head, neck, and abdomen, and has a five-year survival rate of only 40% [78,79]. PCC/PGL is the most heritable of all tumors and carries both germline and somatic mutations in 1 of 20 known genes including metabolic genes. Recently de novo germline mutations in DNMT3A were reported in PCC/PGLs. The mutations occur in the PWWP domain, and result in substitutions Lys299Ile and Arg318Trp (Figure 4A). Although these residues are not the part of the aromatic cage, crystal structure analysis shows that Lys299 interacts with the backbone of Phe303, which stabilizes the interaction of PWWP domain with H3K36me2/3 (Figure 4B). Conversely, Arg318 interacts with the Val35 of the H3 tail, so the substitution Arg318Trp may effect H3 binding. However, future biochemical studies will be needed to show the effect of these mutations on the activity of the enzymes. Methylation profiling of PCC/PGL patients and of HeLa cells carrying CRISPR/Cas9-mediated knock-in of the PGG/PCL DNMT3A mutation show site-specific hypermethylation at homeobox genes, genes involved in dopaminergic neurogenesis, neural crest differentiation, and embryonic morphogenesis. Given that previously known mutations in DNMT3A causing overgrowth syndrome result in genome-wide hypomethylation, the PWWP mutation in PCC/PGL leading to hypermethylation is described as gain-of-function mutation.

Similar DNMT3A heterozygous gain-of-function mutations were recently shown to cause microcephalic dwarfism, a hypocellular disorder of extreme global growth failure, including a reduction in head size and height [80]. The mutations result in substitutions Trp330Arg and Asp333Asn, both of which are located in the DNMT3A PWWP domain (Figure 4C). Both residues are part of the aromatic cage that interacts with H3K36me2/3, and when mutated, the interaction with chromatin is abrogated (Figure 4D) [80]. In contrast to some TBRS mutations, which lead to PWWP domain instability, MD mutations do not affect the stability of this domain [80]. Genome-wide DNA methylation analysis of patients’ fibroblasts showed that similar to PCC/PGL, the majority of differentially methylated regions (DMRs) were hypermethylated compared to wild type samples, and these DMRs were associated with developmental transcription factors and morphogen genes. In the control fibroblasts, these regions were marked by tri-methylation of lysine 27 on histone H3 (H3K27), which is established by the polycomb repressive complex, PRC2, and DNA remains unmethylated [80]. Notably, many of the hypermethylated regions in patient fibroblasts were identified as broad non-methylated islands or differentially methylated valleys in normal cells [81,82,83,84]. Reduced H3K27me3 at these hypermethylated sites occurs despite normal levels of the PRC2 subunit, EZH2 histone methyltransferases in the MD patients. Given that MD DNMT3A variants cannot be targeted to H3K36me3 chromatin domains, they are speculated to methylate transcriptionally repressed regions spuriously, which are otherwise regulated by the PRC2 complex. This phenomenon could be explained by previous observations showing an antagonism between DNA methylation and deposition of H3K27me3. It was further shown that DNA methylation can abrogate binding of the PRC2 protein, SUZ12, to nucleosomes that can impact EZH2 activity at these sites [82,85]. These observations support the speculation that PRC2 binding is impaired by DNA methylation at hypermethylated regions in MD patients. Whereas, loss of EZH2 promotes premature differentiation, loss of DNMT3A increases the stemness of embryonic and progenitor stem cells [86]. The DNMT3A MD variants, however, are proposed to be gain-of-function mutations, which might increase cellular differentiation causing premature depletion of stem/progenitor cell pools. This in turn could affect the growth of tissues and lead to reduced organism size [80].

Comparatively, a single mutation in the PWWP domain of DNMT3B was discovered in patients with ICF syndrome [87]. The mutation S282P also resides in the hydrophobic pocket that interacts with H3K36me3. Earlier data showed that that this mutation leads to decreased or loss of heterochromatin binding of DNMT3B [88]. Later was also shown to affect the interaction of DNMT3B with H3K36me3 at gene bodies [53,89]. Given the complexity imposed by various alternatively spliced isoforms of DNMT3B on its biological activity in various tissues, the S282P variant could have a more diverse effect on DNA methylation landscape compared to similar mutation in DNMT3A. Indeed, the catalytically inactive isoforms of DNMT3B were shown mediate DNA methylation at gene bodies by potentially recruiting DNMT3A to these sites, therefore demonstrating a functional role similar to DNMT3L [90,91]. Global expression and epigenetic profiling of cells derived from ICF patients showed upregulation of genes associated accompanied by loss of SUZ12 binding and H3K27me3. However, in contrast to the growth syndrome MD, these regions remain hypomethylated [92]. This distinctive feature could be due to specific recruitment, protein-protein interactions and activities of DNMT3A and DNMT3B.

## 4. Conclusions and Perspectives

DNA methylation defects in the absence of DNMT mutations have been reported in a plethora of disorders. However, with exception of DNMT3B germline transmitted mutations that cause ICF, disease-causing mutations in DNMT1 and DNMT3A were recently discovered. These mutations cause growth disorders, HSAN1E, ADCA-DN, TBRS and MD that have distinct clinical manifestations.

The ADCA-DN and HSAN1E mutations were mapped to the regions that could potentially impair the interaction of DNMT1-RFTS domain either with the ubiquitin or with the DNA binding region of the MTase domain. The former will render the enzyme in an autoinhibited state, and the latter will lead to an unregulated activity of DNMT1 enzyme making it hyperactive (Figure 1D). Methylome mapping of the patients show aberrant DNA methylation including both widespread hypomethylation and site-specific hypermethylation. This methylation pattern is similar to the one observed in various cancers indicating a similar loss of DNMT1 function, however none of the HSAN1E and ADCA-DN patients develop cancer. It is speculated that in these patients, a gradual loss of DNA methylation over time may cause late onset and progressive neurological disabilities. However, given that post mitotic neurons are terminally differentiated and do not perform maintenance methylation, the effect of RFTS mutations on the activity of DNMT1 could be due to protein misfolding and aggregation [30]. Interestingly, ADCA-DN and HSN1E have some overlapping clinical features typical of mitochondrial diseases, which is supported by biochemical evidence of mitochondrial dysfunction [33,37,93]. Given that presence of methyl cytosine in mtDNA is still debated, it is not clear how DNMT1 mutations can cause mitochondrial dysfunction through its effect on mtDNA methylation. Taken together, these data suggest that DNMT1 mutants could exert their damaging effect through at least two mechanisms including impairment of epigenetic pathways, and cellular stress by the protein misfolding [30,32,33].

Compared to TBRS in which DNMT3A mutations are present in all functional domains of the protein, in MD, the two mutations map only to the PWWP domain. However, in contrast to MD-specific PWWP mutations, the TBRS-specific PWWP mutations affected protein stability resulting in loss of protein function. Based on the observation that the MD variant, DNMT3A Trp330Arg, is unable to interact with H3K36me2/3, it was proposed to be “available” to methylate sites that are normally polycomb repressed [80]. This is consistent with the observation that polycomb repressed developmental genes in ESCs gain DNA methylation during differentiation and are often found hypermethylated in cancer cells [12,83,94]. It is possible that by interacting with PRC2, the DNMT3A Trp330Arg variant is targeted to these regions in MD patients [81,84]. In addition, once the DNA methylation is established, it could potentially block the activity of PRC2 by interfering with the binding of Suz12 [82] and result in stable repression of the developmental genes [85]. Comparatively, in ICF cells, the DNMT3B Ser282Pro variant, which also has impaired binding to H3K36me3, does not methylate PRC2 repressed regions. However, it is obscure how these regions lose PRC2 binding and H3k27me3. Furthermore, mutations of histone methyltransferases, EZH2 and NSD1, cause the Weaver and Sotos overgrowth syndromes, respectively, which supports a critical role of epigenetic regulation in organism size.

The discovery of DNMT mutations in rare diseases has shaped our understanding of the cause and consequence of aberrant DNA methylation in various disorders. It is clear that global hypomethylation, often found in cancers as well as growth disorders, may not be a direct consequence of DNMT loss of function, rather an indirect response to a diseased state. Targeted hypermethylation seems to be direct consequence of aberrant DNMT activity. This is also supported by inconsistency between the biochemical outcomes of DNMT mutations and their effect on genomic methylation. Future studies designed to address the direct effect of DNMT mutations on genomic DNA methylation patterns will help understand the contribution of DNMTs in pathogenesis.
